# Delayed Gut Colonization Shapes Future Allergic Responses in a Murine Model of Atopic Dermatitis

**DOI:** 10.3389/fimmu.2021.650621

**Published:** 2021-03-17

**Authors:** Amalie W. Arildsen, Line F. Zachariassen, Lukasz Krych, Axel K. Hansen, Camilla H. F. Hansen

**Affiliations:** ^1^ Section of Experimental Animal Models, Department of Veterinary and Animal Sciences, Faculty of Health and Medical Sciences, University of Copenhagen, Frederiksberg, Denmark; ^2^ Department of Food Science, Faculty of Science, University of Copenhagen, Frederiksberg, Denmark

**Keywords:** allergy, atopic dermatitis, childhood ezcema, early priming, gut microbiota, immune maturation, rikenellaceae, window of opportunity

## Abstract

Epidemiological studies have long reported that perturbations of the childhood microbiome increase the risk of developing allergies, but a causal relationship with atopic dermatitis remains unclear. Here we colonized germ-free mice at birth or at one or eight week-of-age to investigate the role of prenatal and early postnatal microbial exposure on development of oxozolone-induced dermatitis later in life. We demonstrate that only one week delayed microbial colonization increased IgE levels and the total histological score of the inflamed ear compared to mice colonized throughout life. In parallel, several pro-inflammatory cytokines and chemokines were upregulated in the ear tissue demonstrating an enhanced immunological response following delayed postnatal colonization of the gut. In contrast, sensitivity to oxazolone-induced dermatitis was unaffected by the presence of a maternal microbiota during gestation. Mice colonized at eight week-of-age failed to colonize Rikenellaceae, a group of bacteria previously associated with a high-responding phenotype, and did not develop an immunological response to the same extent as the early colonized mice despite pronounced histopathological manifestations. The study provides proof-of-principle that the first intestinal colonizers of mice pups are crucial for the development of oxazolone-induced dermatitis later in life, and that the status of the maternal microbiota during pregnancy has no influence on the offspring’s allergic immune response. This highlights an important window of opportunity following birth for microbiota-mediated interventions to prevent atopic responses later in life. How long such a window is open may vary between mice and humans considering species differences in the ontogeny of the immune system.

## Introduction

The colonizing commensals in the gastrointestinal tract play an instrumental role in shaping the immune system of the host during infancy (1, 2). Disruption of the colonization process by environmental modulators can lead to long-term changes in the immune system that may increase the newborn’s risk of developing allergic diseases later in life (3, 4). Allergic diseases are one of the primary health problems of our time, and in the last decades the prevalence of asthma, hay fever, food allergies, and eczema has dramatically increased (5). Atopic dermatitis is a chronic eczema affecting up to 20% of children in industrialized regions (6), and the skin disease has a drastic impact on the quality of life of the patient, as it manifests as an intense itch and loss of function in affected areas leading to social stigmatization, self-confidence issues, and sleep loss. Furthermore, atopic dermatitis is often followed by the development of allergic rhinitis and/or asthma (7), i.e. the atopic march. The increasing prevalence of allergic diseases has long been explained by a westernized lifestyle with excessive hygiene, vaccination and widespread use of antibiotics, as well as drastic changes in dietary habits; factors that all modulate the establishment of the early gut microbiome (8, 9). The gut microbiome of children with atopic dermatitis is different from healthy age-matched children (10, 11). In addition, gestational exposure to antibiotics was previously shown to be associated with infant gut microbiota dysbiosis (12) and childhood eczema (13), and prenatal microbial exposure to agricultural environment has also proven to influence development of allergy later in life (14), thus, highlighting the importance of understanding long-term consequences of both prenatal and postnatal microbiome-host interactions. In particular the studies demonstrating an association between the early gut microbial composition and the onset and severity of atopic dermatitis later in life point towards a possible causal role of the gut microbiota (15), though the link remain controversial due to conflicting results in human observational and interventional studies (16), and calls for proof-of-concept experiments. Rodent studies support a causal role of the gut microbiota, as high- and low responding phenotypes in mice with oxazolone-induced atopic dermatitis are transferable by transplantation of their microbiomes to germ-free mice (17). Manipulation of the infant gut microbiota in mice with prebiotics has also shown beneficial effects on atopic disease manifestations later in life (18), supportive of the development of new and more efficient microbiota-mediated prevention strategies early in life in children at risk of atopic dermatitis.

Experimental data from germ-free mice have previously demonstrated the importance of a diverse gut microbiota colonizing early in life in preventing abnormally high serum IgE levels (19). Newborn pups that are colonized late do not restore IgE levels. Similarly, other abnormalities in the immune system of germ-free mice are not reversed upon later colonization (2), suggesting a critical window in time following birth in which perturbations of host-microbiota interactions lead to irreversible aberrant immune responses with potentially profound impact on future health. In the present study, we sought to clarify the long-term consequences on oxazolone-induced dermatitis following an early short-term disruption of the gastrointestinal colonization by delaying microbial exposure in newborn pups.

## Materials and Methods

### Ethics

The experiment complied with the Danish Act on Animal Experimentation which implements the Directive 2010/63/EU on the Protection of Animals used for Scientific Purposes. It was approved by the Danish Animal Experiments Inspectorate at Ministry of Environment and Food (License number: 2012-15-2934-00399).

### Animals

**Figure 1:**
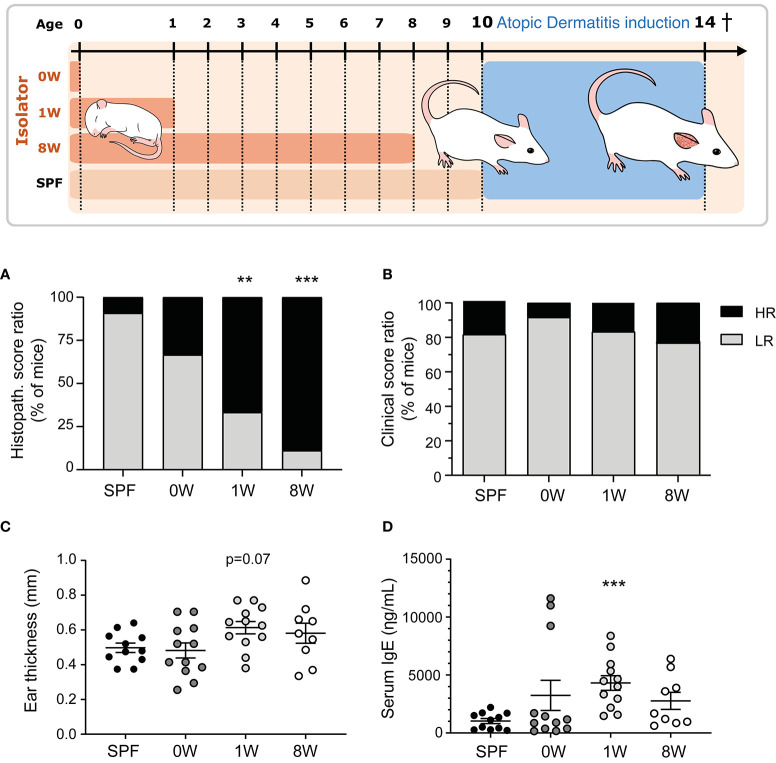
Sensitivity to oxazolone-induced dermatitis in SPF and ex-germ-free mice. The experimental setup is outlined with three germ-free groups colonized at birth (0W, *n*=12), 1 week-of-age (1W, *n*=12), or 8 week-of-age (8W, *n*=9) and one microbiota-associated SPF bred control group (SPF, *n*=11). After 10 weeks-of-age, the mice were sensitized with 0.8% oxazolone before they were challenged ten times with 0.2% oxazolone solution on the right ear. The mice were euthanized at 14 weeks of age after the last challenge and sampled for further analyses. **(A)** HE stained cross sections of the inflamed ears were scored blindly in all mice. The percentages of mice expressing a high (total score >8.5) or low (total score ≤8.5) responding phenotype are shown. **(B)** The degree of oxazolone-induced dermatitis was clinically scored by two blinded persons independently in all mice when euthanized. The percentages of mice expressing a high (average total score >7.5) and low (average total score ≤7.5) phenotype are shown. **(C)** Ear thickness was measured twice on anaesthetized mice immediately before euthanization. Mean and SEM are shown. **(D)** Serum IgE was measured with ELISA in all mice. Mean and SEM are shown. ** indicate *p* < 0.01, *** indicate *p* < 0.001. All *p* -values below 0.1 are indicated.

Germ-free Swiss Webster mice (Taconic, Germantown, NY) were housed in our AAALAC accredited germ-free facility (University of Copenhagen, Frederiksberg, Denmark) in HEPA-ventilated isolators (PFI systems, Milton Keynes, UK; pressure 110 pascal, 23°C) with free access to an irradiated Altromin 1324 diet (Brogaarden, Lynge, Denmark) and sterile water. Germ-free status was tested both by culturing and PCR methods (17). Breeding pairs were transferred a few weeks before mating to our AAALAC accredited barrier protected facility (room temperature 22 ± 2°C, relative humidity 55% ± 10%, air changes 15-20 times per hour, lighting interval 06.00am - 06.00pm) and their female pups (*n* = 11) were used as control mice that were fully colonized throughout life. Other germ-free female pups were transferred to the barrier facility at birth (*n* = 12), one week (*n* = 12) or eight weeks (*n* = 9) after birth (together with the dams of the pre-weaned pups) and housed in open cages without filter lids and given dirty bedding from the cages containing the barrier bred control mice. The experimental groups are illustrated in **Figure 1**. Female pups from at least three litters were included in each group and fed the same diet as the isolator mice.

### Induction of Atopic Dermatitis

At 10 weeks of age, all mice were sensitized once on the medial and lateral sides of the right ear with 20 µL oxazolone solution containing 0.8% oxazolone (w/v) (4-ethoxymethylene-2-phenyl-2-oxazolin-5-one, E0753, Sigma-Aldrich, St Louis, MO) dissolved 4:1 in acetone (Emsure, MerckChemicals, Darmstadt, Germany) and oil (Organic extra virgin olive oil, Svansø, Scandic Food A/S, Vejle, Denmark) applied with a pipette (Day -7). After one week the mice were challenged on the medial and lateral sides of the right ear with 20µL 0.2% oxazolone solution dissolved 2:1 in acetone and oil on days 0, 3, 5, 7, 10, 12, 14, 16, 18, and 20. Both sensitization and challenges were performed on unanaesthetized animals and it should therefore be expected that the mice will lick the ears and swallow parts of the oxazolone solution. All mice were killed on day 21 at 14 weeks of age (**Figure 1**).

### Clinical Scores

Dermatitis was scored blindly at euthanasia by two independent persons. The inflammation was evaluated on (i) redness, (ii) thickening, (iii) excoriation and erosion, and (iv) incrustation and xerosis. Scoring grades were assigned for each of the four parameters as follows: 0 = no sign; 1 = mild; 2 = moderate; 3 = severe, resulting in a total score up to 12. The average total score was calculated for each animal. Ear thickness was measured on the day of sensitization on unanaesthetized mice and immediately before euthanasia on anaesthetized mice with a micrometer (Mitutoyo Low Force Caliper Series 573, Aurora, IL); each measurement was repeated twice.

### Histology

Paraffin blocks of formalin fixed ear tissue biopsies were cut at 2µm cross sections and stained with hematoxylin and eosin (HE). Histopathological scoring was done blinded by two independent persons based on epidermal spongiosis, dermal hyperplasia, mast cell infiltration, neutrophil infiltration, and fibroplasia that were each given a score of 0 = none, 1 = slight, 2 = distinct, or 3 = marked, resulting in a total score up to 15.

### Serum IgE

Serum IgE levels were measured using Mouse IgE ELISA Kit (Bethyl Laboratories, Montgomery, TX) according to the standard protocol using a 1:20 dilution of the samples. Absorbance was measured and analyzed (PowerWave x Microplate Spectrophotometer and KC4 v3.4, Rev 21, Bio-Tek Instruments INC, Winooski, VT).

### Ear Tissue Cytokines

Ear biopsies were weighed and homogenized by blending (POLYTRON PT1200E Manual Disperser, Kinematica AG, Luzern, Switzerland) in 300µL lysis buffer (Meso Scale Discovery, Rockville, MD) and stored at 4˚C. Levels (within the given detection ranges) of IFN-γ (0.16-644 pg/ml), IL-1β (0.37-1510 pg/ml), IL-2 (0.73-2980 pg/ml), IL-4 (0.41-1617 pg/ml), IL-5 (0.23-947 pg/ml), IL-6 (1.22-4990 pg/ml), CXCL1 (0.46-1870 pg/ml), IL-10 (0.86-3530 pg/ml), IL-12p70 (7.42-30400 pg/ml), and TNF (0.16-662 pg/ml) were measured using MSD MULTI-SPOT Assay System, V-PLEX Proinflammatory Panel 1 mouse assay (Mesoscale Discovery). Levels of IL-17A (0.51-2070 pg/ml), IL-17C (9.16-37500 pg/ml), IL-17F (10.8-44100 pg/ml), IL-25 (2.83-11600 pg/ml), IL-21 (6.86-28100 pg/ml), IL-22 (0.42-1730 pg/ml), IL-23 (4.71-19300 pg/ml), IL-31 (14.8-60500 pg/ml), and IL-33 (7.89-32300 pg/ml) were measured using a MSD U-PLEX Th17 Combo 1 mouse assay, however only IL-33 was above the lower detection limit. Both assays were read on a MESO QuickPlex SQ120 and analyzed by the standard software Discovery Workbench v4 (Meso Scale Discovery). The cytokines and chemokine levels were normalized to total protein levels measured with Pierce Detergent Compatible Bradford Assay kit according to manufacturer’s protocol.

### Gut Microbiota Analysis

The fecal microbiota composition was determined for the mice at 10 weeks of age before sensitization. DNA was extracted with QIAamp DNA stool mini kit (Qiagen, Hilden, Germany), and the bacterial community relative abundance was evaluated using nanopore based sequencing of the near full 16S rRNA gene amplicon using MinION (Oxford Nanopore Technologies, Oxford, UK) as previously described (20). The genomic DNA was extracted using Bead-Beat Micro AX Gravity Kit mod1 (A&A Biotechnology, Gdynia, Poland) according to the manufacturer’s instruction. DNA concentration and purity was measured using NanoDrop ND-1000 spectrophotometer (Saveen & Werner AB, Limhamn, Sweden) and the extracted DNA was diluted to 10 ng/µL prior to library preparation. Near full-length 16S rRNA gene amplicons were amplified and sequenced with ONT targeting V1-V8 hypervariable region using following primers: ON.16S_27Fa: GTCTCGTGGG CTCGGAGATG TGTATATAGA TCGCAGAGTT TGATYMTGGCTCAG; ON.16S_27Fb: GTCTCGTGGG CTCGGAGATG TGTATATAGA TCGCAGAGTT TGATCCTGGCTTAG and ONT_1540_R: GTCTCGTGGG CTCGGAGATG TGTATACTCT CTATTACGGY TACCTTGTTACGACT. A custom designed barcoding system was developed to tag encode up to 96 samples during the second round of PCR. The 1^st^ and 2^nd^ PCR primer sequences are given in Hui et al. (20). The PCR1 reaction mix contained 5µl of PCRBIO buffer and 0.25 µL PCRBIO HiFi polymerase (PCR Biosystems Ltd, London, UK), 1 µl of primers mix (5 µM of ON.16S_27Fa a and ON.16S_27Fab, and 10 µM of ONT_1540_R), 5 µl of genomic DNA (~10ng/ul) and nuclease-free water to a total value of 25 µl. The PCR thermal conditions were as follows: denaturation at 95°C for 5 min; 33 cycles of 95°C for 20 s, 55°C for 20 s and 72°C for 45 s; followed by final elongation at 72°C for 4 min. PCR products were verified by agarose gel electrophoresis and then subjected for barcoding (PCR2). The PCR2 mix was composed of 5 µL PCRBIO buffer, 0.25 µL PCRBIO HiFi polymerase (PCR Biosystems Ltd), 2 µL of barcode primers (5 µM), 1 µL of PCR1 template and DEPC water up to 25 µL. The PCR2 thermal conditions were as follows: denaturation at 95°C for 2 min; 13 cycles of 95°C for 20 s, 55°C for 20 s, 72°C for 40 s; final elongation at 72°C for 4 min. The final PCR products were purified using AMPure XP beads (Beckman Coulter Genomic, Danvers, MA) and pooled in equimolar concentrations. The pooled barcoded amplicons were subjected to 1D genomic DNA by ligation protocol (SQK-LSK109) to complete library preparation for MinION sequencing. ~0.2 μg of amplicons were used for the initial step of end-prep. ~40ng of prepared amplicon library was loaded on a R9.4.1 flow cell.

Data generated by MinION were collected using MinKnow software v19.06.8 (https://nanoporetech.com). The Guppy v3.2.2 basecalling toolkit was used to base call raw fast5 to fastq (https://nanoporetech.com). Porechop v0.2.2 was used for adapter trimming and sample demultiplexing (https://github.com/rrwick/Porechop). The Porechop adapter list was (adapters.py) edited accordingly and is given in Hui et al., 2020 (20). Sequences containing quality scores (fastq files) were quality corrected using NanoFilt (q ≥ 10; read length > 1Kb). Taxonomy assignment of quality corrected reads against Greengenes (13.8) database was conducted using uclast method implemented in parallel_assign_taxonomy_uclust.py Qiime (v1.9.1). The uclust settings were tuned on mock communities (–similarity 0.8; min_consensus_fraction 0.51) assuring annotations to the lowest taxonomic level with no false positive annotations. The settings allow us to treat individual Oxford Nanopore Technologies - Amplicon Sequence Variant (ONT-ASV) as individual “seeds”. Only reads classified to at least phylum level were subjected for further analysis.

The generated abundance table was further analysed using Qiime2 (v2018.11) bioinformatic platform (21). Alpha (observed species index) and beta diversity (PCoA based on Bray-Curtis and Jaccard distance matrices) were calculated using rarefaction to 13,000 reads per sample, which was 80% of the most indigent sample in the dataset. Permutational multivariate analysis of variance (PERMANOVA) was used to test differences between categories based on Bray-Curtis and Jaccard distance matrices. Analysis of compositions of microbiomes (ANCOM) was used to find compositional differences in the bacterial community (22).

### Statistics

GraphPad Prism version 6.03 (GraphPad Software, San Diego, CA) was used for statistical analysis and *P*-values less than 0.05 were considered significant and *P*-values less than 0.1 were stated as tendencies. Gaussian distribution tests (D’Agostino & Pearson omnibus, or Shapiro-Wilk normality test if n<5) were applied to all quantitative data. Data were analyzed using one-way ANOVA with Dunnett’s multiple comparison test comparing the mean of every group to the SPF group, or non-parametric Kruskal-Wallis test with Dunn’s multiple comparison test for data that did not assume Gaussian distribution. Welch’s correction was included if variances were unequal by Brown-Forsythe test. For clinical and histopathology score, a point of reference equal to the 75% percentile of frequency distribution in the SPF group divided the animals in on/below (≤) or above (>) point of reference. These dichotomous data were analyzed with Chi-square (χ^2^-test). Principal Components Analysis on host immune parameters was conducted using stats R package (v3.5.1).

## Results and Discussion

### Postnatal, but Not Prenatal, Germ-Free Life Exacerbated Oxazolone-Induced Dermatitis

Here we demonstrate that the first intestinal colonizers regulate the development of oxazolone-induced dermatitis. One (1W) or eight weeks (8W) germ-free life immediately after birth increased sensitivity to oxazolone-induced dermatitis compared to SPF bred mice based on histological evaluation of spongiosis, hyperplasia, fibroplasia, and mast cell and neutrophil infiltration (**Figure 1A** and **Supplementary Figure 1**). Only one mouse in the SPF group received a high histopathological score above the upper 75% percentile, compared to 8 out of 12 and 8 out of 9 in the 1W and 8W germ-free groups respectively (**Supplementary Figure 2**). In contrast, we show that the maternal microbiota during gestation has no long-term impact on oxazolone-induced dermatitis in the pups. The mice that were germ-free in utero (0W) had similarly low numbers (4 out of 12) of high-responders as the SPF mice. No differences were observed in the clinical skin inflammation score (**Figure 1B** and **Supplementary Figure 3**). The parameter is however not very sensitive to variation as rather large differences in the macroscopic manifestations are necessary for the mice to receive different scores (**Supplementary Figure 3**), which may explain why it did not correlate to the rest of the parameters as described below (**Figure 3**). The oxazolone treatment significantly increased ear thickness on the treated ears from an average on 187µm ± 42µm on Day -7 to 531µm ± 14µm on Day 21 (mean ± s.d.; *p* < 0.001) demonstrating the successful induction of dermatitis (**Supplementary Figure 3**). There was a strong tendency for increased ear thickness measurements (**Figure 1C**, *p* = 0.07), and significantly higher IgE levels (**Figure 1D**) in the 1W germ-free group compared to the SPF bred mice. This was only evident in the 1W group as the 0W and 8W groups were not significantly different from the SPF mice.

### A Short-Term, but Not Long-Term, Delayed Colonization Enhanced the Local Immunological Response

**Figure 2:**
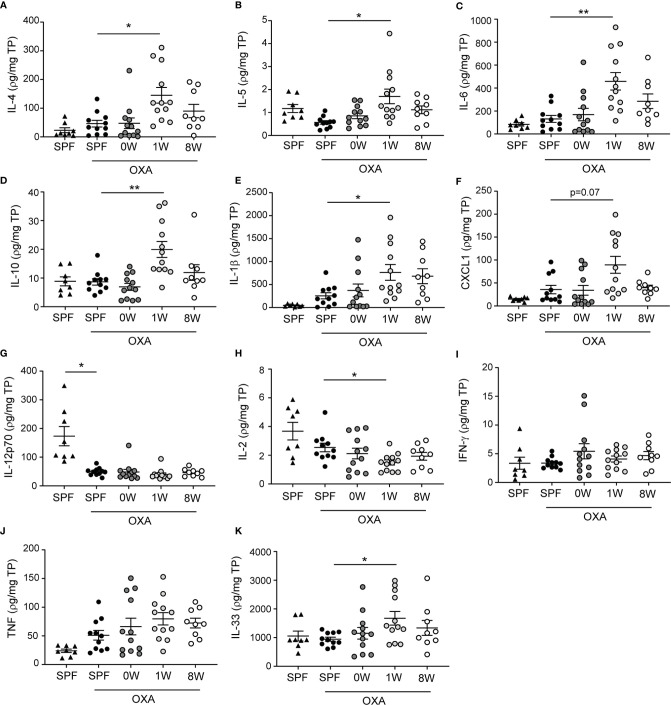
Ear tissue cytokine levels were upregulated after a short-term delayed colonization. **(A–K**) Cytokines were measured with two mesoscale multiplex kit in punch biopsies sampled from the inflamed ear (SPF, *n*=11, 0W, *n*=12, 1W, *n*=12, 8W, *n*=9) and from untreated ears of SPF mice (*n*=6) for comparison. The levels of each cytokine and chemokine were normalized to the total protein concentration per sample. Mean and SEM are shown for each cytokine. * indicates *p* < 0.05, ** indicate *p* < 0.01. All *p* -values below 0.1 are indicated.

The strong phenotype of mice being germ-free in their first week of life was also observed in ear tissue cytokine levels. Keratinocytes release inflammatory mediators including IL-33, IL-25, and TSLP in patients with atopic dermatitis which activates a type 2 immune response characterized by IL-4, IL-5, and IL-13 producing T cells, and high IgE antibody titers. In the mice, IL-33, IL-4, IL-5, IL-6, and IL-10 as well as the neutrophil activator IL-1β and chemoattractant CXCL1 were all up-regulated significantly in the 1W group compared to SPF mice (**Figure 2**). Large amounts of IL-6 is also produced by CD4^+^ T cells in lesions of human atopic dermatitis (23), as well as IL-10 which is overexpressed in atopic dermatitis (24) and may act both pro- and anti-inflammatory as an inducer of the Th2 response and an inhibitor of IL-17 driven neutrophil infiltrations (25). No significant differences were found in TNF or Th1 associated cytokines IFN-γ and IL-12p70 between the groups, except for very low levels of IL-12p70 in all oxazolone treated ears compared to the untreated ears (**Figure 2**). IL-12p70 is naturally inhibited by the Th2 immune response induced by repeated oxazolone treatments. The lymphoproliferating cytokine IL-2 was the only down-regulated cytokine in the 1W group compared to the SPF group, which is similar to findings in human patients with atopic dermatitis in whom IL-2 is inversely correlated to the severity of disease (26). None of the differences in the inflammatory mediators measured were evident in the 0W group, supporting the histological findings that if the mice were germ-free period only during gestation, it had no effect on the development of dermatitis.

**Figure 3:**
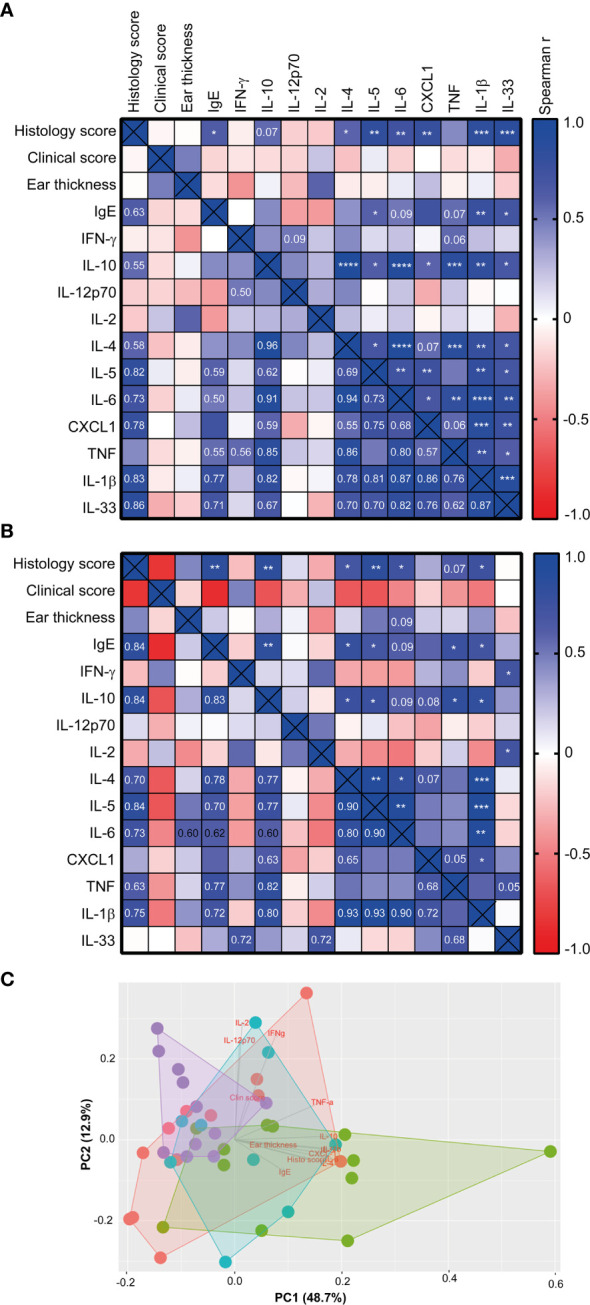
The histology score correlated with the immune response of high-responding mice Heatmaps illustrating significant correlations between phenotypic disease markers used to characterize disease development and immunological responses in the 1W group (*n*=12) **(A)**, and in the 8W group **(B)**. P-values are indicated in the upper right half of the heatmaps and the r^2^ values are given in the lower left halfs for correlations with p-values < 0.1. The color codes indicate the positive or negative r value (correlation). **(C)** Principal component analysis plot based on the phenotypic disease and immune markers listed in **(A)**, showing clusters of SPF (*n*=11, purple), 0W (*n*=12, red), 1W (*n*=12, green) and 8W (*n*=9, blue) mice. * indicates *p* < 0.05, ** indicate *p* < 0.01, *** indicate *p* < 0.001, **** indicate *p* < 0.0001.

When correlating the different host parameters in the 1W group, it was obvious that it was indeed the same mice that had the highest histopathological score, which also had the highest IgE and ear tissue cytokine levels (**Figure 3A**). Nonetheless, even though they exhibited clear pathological changes (**Figure 1A** and **Supplemantary Figure 1**) and increase in ear thickness, none of the oxazolone treated SPF mice showed significantly upregulated cytokines or chemokines in the inflamed ears compared to the untreated ears (**Figure 2**). This indicates that changes in the cytokine levels may be more discrete than the effect of oxazolone on the microscopic findings, and, therefore, larger group sizes may be necessary to increase the power and detect such differences, which also seems to be the case for the 8W group in which none of the differences in cytokine or chemokine levels were significant, although some of the mice showed upregulation of some of the cytokines. A less conservative t-test between the SPF and 8W group did indeed find significant higher levels of IL-1β, IL-5, IL-6 and a tendency for IL-4 (*p* = 0.09) in the 8W group (not shown), and significant correlations between the pathological findings and inflammatory mediators in the 8W group (**Figure 3B**) indicate that the discrepancy between the two kind of parameters is in fact a matter of power. Taken together, comparison of the groups by principal component analysis based on all pathological and immunological parameters demonstrated that only the 1W group significantly differed from the SPF mice (*p* < 0.01 on PC1, **Figure 3C**), suggesting an overall reduced response in the 8W group compared to the 1W group.

### Rikenellaceae Failed to Establish in Mice Colonized Late

**Figure 4:**
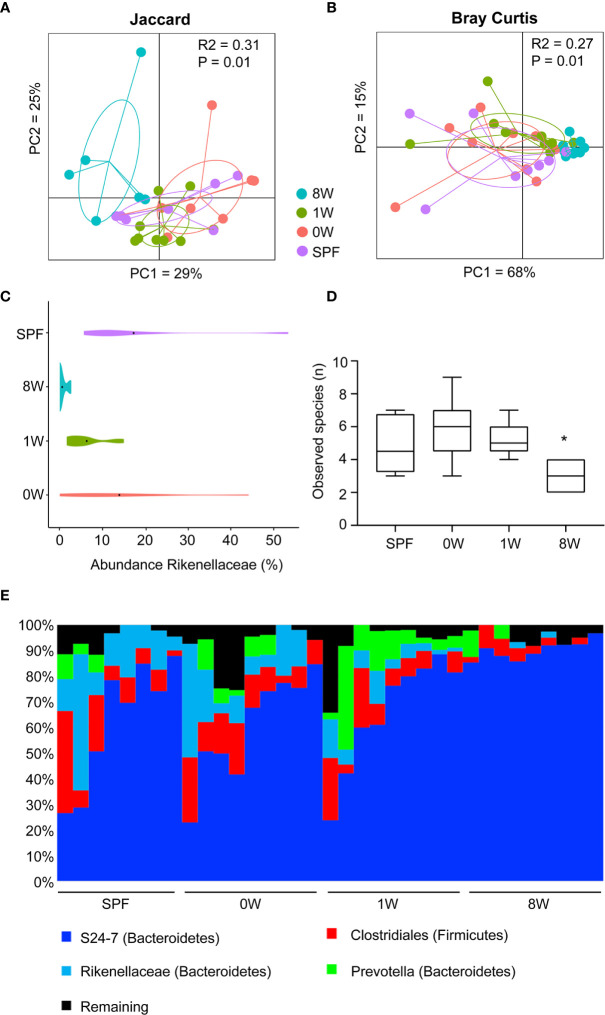
Late colonization alters the gut microbiota composition. Principal coordinates analysis plot of nanopore based sequencing of the near full 16S rRNA gene amplicon based on **(A)** Jaccard (unweighted) and **(B)** Bray-Curtis (weighted) distance matrices as indicated. The plots illustrate feces samples from SPF (*n*=8, purple), 0W (*n*=9, red), 1W (*n*=9, green) and 8W (*n*=9, blue) mice before sensitization with oxazolone at 10 week-of-age. **(C)** Violin plot illustrating the abundance of the only taxa significantly different between the groups, the family Rikenellaceae. **(D)** Alpha diversity plot showing the observed species index based on species level summarized ASV table. **(E)** Chart illustrating the distribution of the main taxa colonizing the gut. * indicates *p* < 0.05.

To investigate whether the host responses were in fact determined by delayed colonization or whether different microbiome compositions post colonization could explain the discrepancy between a short and long-term delayed colonization, 16S sequencing of fecal pellets sampled before sensitization was performed. Principal coordinates analysis plot based on Jaccard and Bray Curtis distance matrices showed that the 8W germ-free group clustered significantly different from the SPF, 0W, and 1W groups (**Figures 4A, B**). The difference was based on members belonging to the Rikenellaceae family that failed to colonize the 8W mice (**Figure 4C**). Interestingly, this family has previously been associated with a high-responding phenotype (17), and it is thus possible that the failure of Rikenellaceae bacteria to colonize the intestine of the 8W germ-free mice may have resulted in a milder inflammatory response to oxazolone in contrast to the mice in the 1W germ-free group. This was also evident in the alpha diversity plot where only the 8W group had a significantly lower diversity due to the lack of Rikenellaceae compared to SPF bred mice (**Figure 4D**). Especially at weaning, the gastrointestinal tract experiences an immense functional maturation with introduction of solid feed, including changes in the functional properties of the gut barrier and mucosal immunity that are important determinants for the gut microbiota colonization process in addition to which nutrients are available (27). It is therefore not so strange that colonization post weaning could result in different succession for certain bacteria. There was however no other significant differences between the groups in the distribution of the main taxa colonizing the gut (**Figure 4E**), and there were no significant correlations between specific bacterial taxa, including Rikenellaceae, and the different host parameters. Hence, the increased sensitivity to oxazolone-induced dermatitis in the 1W group was solely due to the delayed colonization and not differences in the gut microbiota composition. It is however possible that metagenomic analysis of the entire gene pool would have enabled identification of microbial functions that could correlate to the severity of dermatitis. If more bacteria have overlapping functions, such correlations would not expectedly appear in the 16S analysis.

### Concluding Remarks

Prenatal and postnatal microbial exposure profoundly impacts development of the host immune system (2, 28). Delayed or altered colonization patterns due to e.g. postpartum antibiotic treatment, sterile settings associated with cesarean section or different types of feeding prevent the natural IgE inhibition that follows microbial stimuli from a fully colonized infant gut (19). Here we show the long-term consequences of lacking such appropriate stimuli early in life as delayed colonization exaggerated oxazolone-induced dermatitis even though the delay only lasted for a very short period of time, while the effect was reduced after a longer period of delayed colonization. The late colonization of the adult gut resulted in a different gut microbiota composition that may by itself affect the immunological response and severity of disease. The effect of delayed colonization on oxazolone-induced dermatitis was not detected in mice colonized upon birth, and in utero exposures including maternal stress, gestational diabetes, antibiotic treatment and other environmental factors that can affect the maternal microbiota and which has been associated with a high risk of developing childhood eczema (9), may as such only play a long-term role for immune maturation and atopic disease if they also disturb the intestinal colonization of the infant gut following birth. Thus, we find that the colonization pattern of the very first colonizers is crucial for the development of atopic dermatitis. In perspective, it is important to note that differences between pre- and postnatal immune development in humans and mice could influence the translatability of the study. The immune system of mice is less mature than in humans in the first week of life (29), and may as such be more dependent on the first microbial stimuli upon birth to develop a strong anti-inflammatory immune response that can prevent an excessive Th2 response later in life. However, considering how very early life events in humans that are known to change the initial microbiota colonization process of the gut can increase the children’s risk of developing childhood eczema (30, 31), the current animal study highlights the causal role the gut microbiota can play in such observed associations, and the potential for implementing early microbiota-directed prevention strategies in infants predisposed of developing atopic disorders.

## Data Availability Statement

The datasets presented in this study can be found in online repositories. The names of the repository/repositories and accession number(s) can be found below: NCBI, accession PRJNA699224.

## Ethics Statement

The animal study was reviewed and approved by Danish Animal Experiments Inspectorate at Ministry of Environment and Food (License number: 2012-15-2934-00399).

## Author Contributions

The author**’**s responsibilities were as follows: AA, AH, and CH conceived and designed the experiments. AA, LZ, and LK performed the experiments. AA, AH, and CH analyzed and interpreted the data, and CH wrote the manuscript. All authors contributed to the article and approved the submitted version.

## Conflict of Interest

The authors declare that the research was conducted in the absence of any commercial or financial relationships that could be construed as a potential conflict of interest.
