# Supplementary figures and images for: Delayed Gut Colonization Shapes Future Allergic Responses in a Murine Model of Atopic Dermatitis
Source: Front Immunol. 2021 Mar 17;12:650621. doi: 10.3389/fimmu.2021.650621 (PMC8010263; doi:10.3389/fimmu.2021.650621)

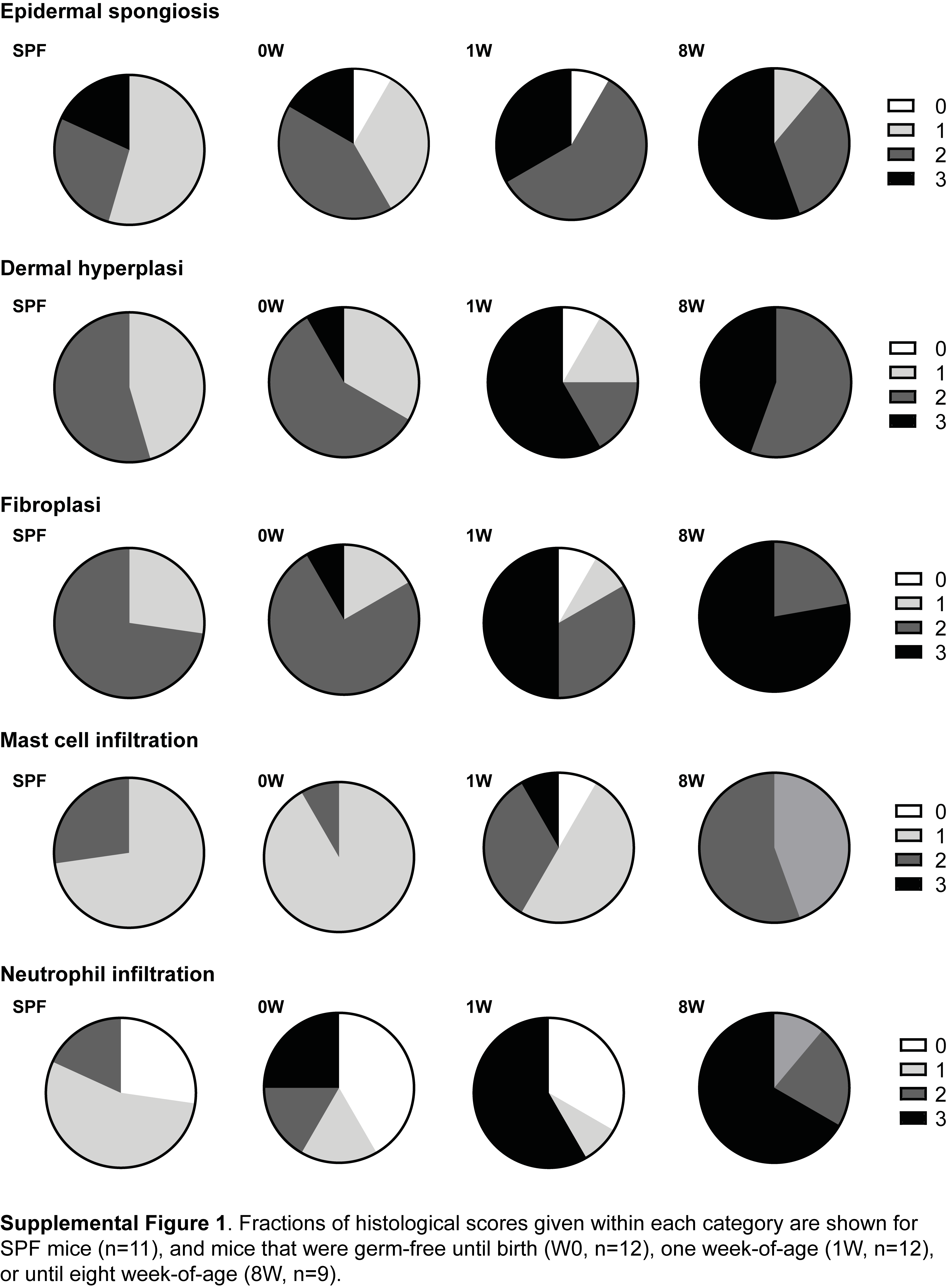

Supplement: Supplementary file 1 [file Image_1.tif]

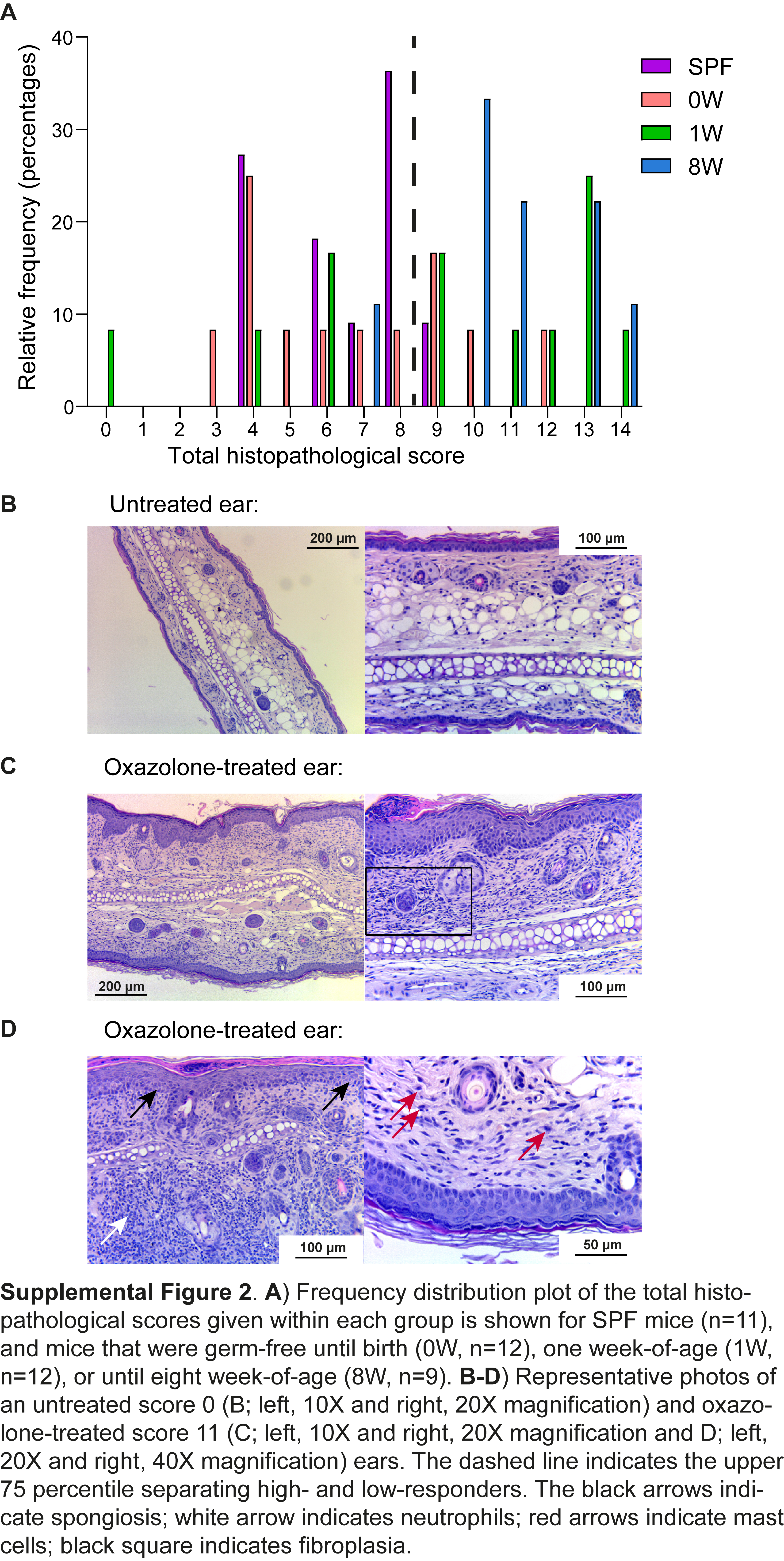

Supplement: Supplementary file 2 [file Image_2.tif]

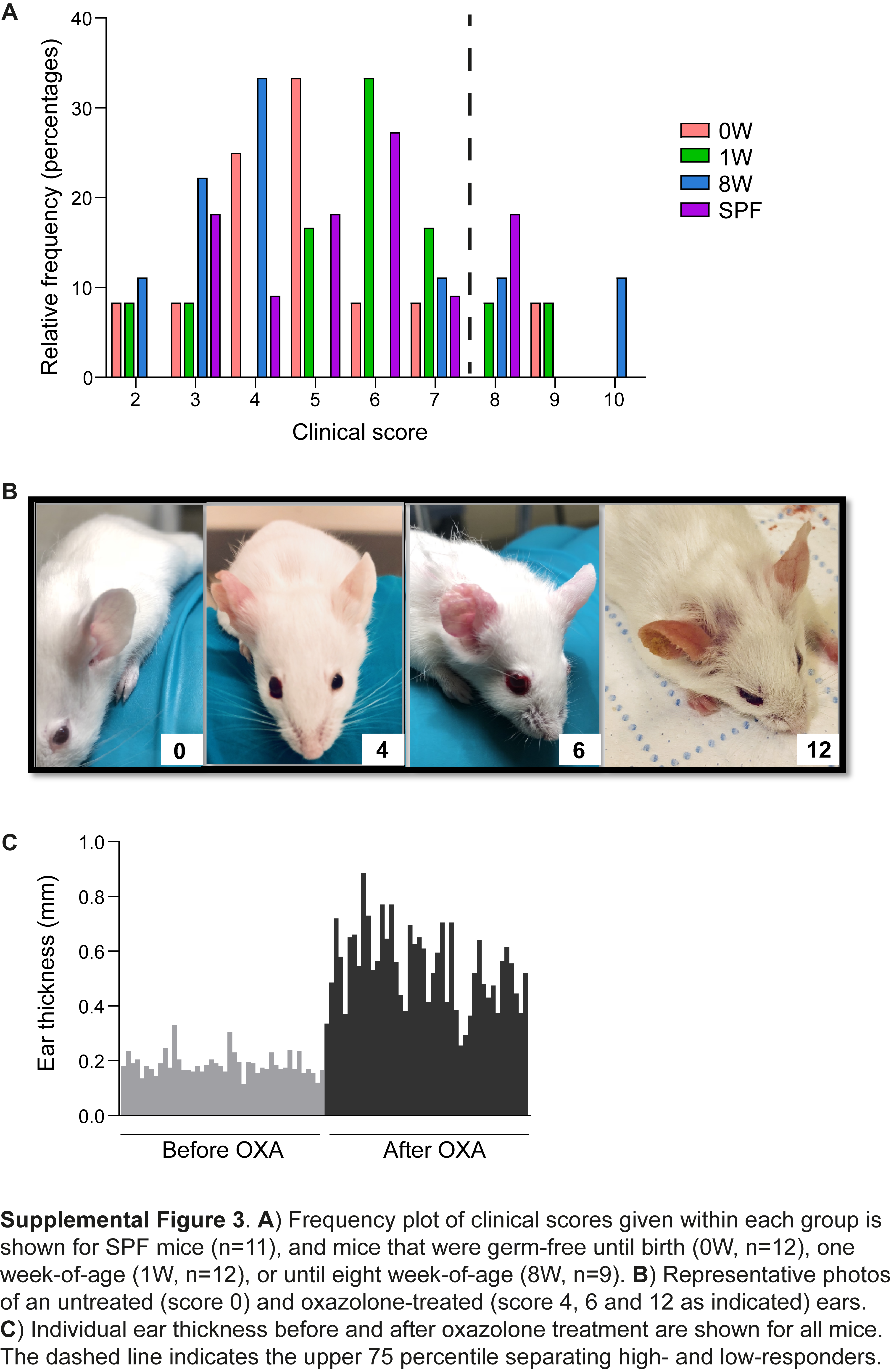

Supplement: Supplementary file 3 [file Image_3.tif]
